# Report on Gynæcology

**Published:** 1874-03

**Authors:** A. Reeves Jackson

**Affiliations:** Lecturer on Gynæcology, Rush Medical College


ARTICLE I.—*Report on Gynæcology.* By A. REEVES JACKSON, M.D., Lecturer on Gynæcology, Rush Medical College.

1. Anticipation of Post-Partum Hemorrhage. DR. EWING WHITTLE. (*British Medical Journal*, Sept. 27, 1873. *American Journal of the Med. Sci.*, January, 1874.)

2. On the Systematic Examination of the Abdomen, with a view to Rectifying Malpositions of the Fœtus during Labor. By ARTHUR EDIS, M.D. (*Med. Times and Gazette*, Jan. 11. *Half-Yearly Abs. of the Medical Sciences*, July, 1873.)

3. Fetal Physical Diagnosis. DR. HUNTER. (*The American Practitioner*, Dec., 1873. *Obstetrical Journal of Great Britain and Ireland*, *American Supplement*, Dec., 1873.)

4. Hydrate of Chloral in Puerperal Convulsions. By ALEXANDER MILNE, M.D. (*Transactions of the Edinburgh Obstetrical Society*, 1872.)

5. Hydrate of Chloral and Bleeding in Puerperal Convulsions. By P. J. ROEBUCK, M.D. (*American Journal of the Medical Sciences*, Jan., 1874.)

6. New Operation for Contraction of the Vaginal Orifice of the Neck of the Uterus. By DR. V. SABOYA. (*Revista Medico Rio de Janeiro*, Aug. 10, 1873. *American Journal of Medical Sciences*, January, 1874.)

7. Clinical Lectures on Diseases of Females. By T. GALLARD. Paris, 1873.

1. Dr. Whittle states that post-partum hemorrhage may be diagnosed in advance, and being diagnosed, may be prevented. He says that hemorrhage may be expected when the pains during parturition are "strong and quick; they do not gradually culminate into a strong pain and subside again, but they are sharp, quick, and cease almost suddenly; and the intervals between the pains are long in proportion to the length of the pains." In normal labor, for one or two hours before its completion, the length of the pains is about one-third of that of the interval; that is, if the pains last from fifty to sixty seconds each, the intervals will average about three minutes, or a little less. Now, according to Dr. Whittle, if the pains have the sharp, quick character described, and last only from forty to fifty seconds, with intervals of five or six minutes, hemorrhage will be sure to occur after the delivery is completed, even though the labor may proceed steadily, and the head advance with every pain. The explanation is this: the uterus contracts sharply, and then becomes fully relaxed; after the delivery, a relaxation follows; one or two sharp pains expel the placenta with a gush of blood, and the uterus is again relaxed, the same tendency continuing which existed before the birth of the child.

The means used and recommended by Dr. W. for the prevention of the flooding, consist in altering the character of the pains—making them longer and the intervals shorter, thus causing them to approximate those of natural labor. This is effected by the use of ergot. Dr. W. gives, so soon as the os is dilated, a full dose of the drug, and if this does not produce the desired effect at the end of an hour, he repeats it. "In dealing with primiparæ, caution is required, first, not to administer ergot until the soft parts are pretty well dilated, as well as the os uteri; and the drug should be administered in much smaller doses, as it sometimes acts with unusual energy in primiparæ. Generally, in about twenty minutes or half an hour after the ergot has been administered, the pains increase in length and frequency, and when the labor is over, the uterus maintains a good contraction."

2. Dr. Edis advocates the systematic examination of the abdomen either prior to, or during the early stage of labor, with a view to rectifying malpositions of the fœtus by external manipulation. Dr. Edis expressed the belief that the universal adoption of this expedient would tend materially to diminish the large mortality which occurs from accidents of childbirth. The process of external version was described as very simple. With the patient on her back, and the knees drawn up, a little experience will soon enable the practitioner to detect the position of the fœtus; and by turning the patient on her side, and making pressure and counter-pressure on the head and breech, the correction of any abnormal position may be readily accomplished. Every physician should consider it his duty to examine carefully the position of the fœtus in utero on first visiting his patient in labor. The diagnosis of the fœtal position can be made out before the membranes are ruptured, and version can be performed even before labor begins. Version by this method can be made during labor with much less danger to the mother and the child than is possible by any other mode.

Dr. Barnes states that a head presentation is the type of a natural labor, and it follows that to obtain a head presentation is the great end to be accomplished by art. By external manipulation, cephalic version can be performed as readily as either podalic or pelvic version. The advantages of this method can be at once appreciated where interference at a very early stage of labor may be desirable, as in cases of placenta prævia, accidental hemorrhage, and convulsions.

3. Dr. Hunter gives the history of three cases in which cephalic version was performed by external manipulation before the rupture of the membranes. In two of these cases the malposition was shoulder presentation.

Dr. Hunter concludes that in cases where the malposition is rectified prior to labor, "it is necessary to re-examine the case at intervals to see that the fœtus does not resume its old position, this being the more imperative during the first part of labor. I believe firmly that were every case examined prior to the rupture of the membranes, the abnormal presentation recognized, and the proper measures instituted, there would be no necessity for the record of an unnatural labor."

4. Dr. Milne reports a case of a woman in labor with her fourth child, who, as the labor was progressing normally towards conclusion, was frightened by the noise of a falling body, and went into a convulsion. The child was born, but the fits still continued at short intervals. Sixty grains of hydrate of chloral were given, and in about fifty minutes the eclampsia ceased, and the patient fell into a heavy sleep which lasted eight hours. She awakened confused, but free from headache or sickness, and made a good recovery.

5. Dr. Roebuck's case is one of a woman twenty-two years of age, who was advanced eight and a half months in her first pregnancy, and who, without any premonitory symptoms, was attacked with violent convulsions. Sixteen ounces of blood were taken after the third fit, and a half grain of sulphate of morphia administered; vomiting soon followed; pulse eighty per minute. The bleeding was repeated in three hours, and another half grain of morphia given. Vomiting again followed, and there was slight abatement of the spasms; pulse eighty. At the end of five hours after the onset of the attack, the patient was entirely unconscious, the convulsions returning every twenty or thirty minutes, and lasting from ten to fifteen minutes. Pulse still eighty, and the pupils fixed.

Although the vagina was well lubricated and flaccid, there was no dilatation of the os uteri, no evidence of uterine contractions, and the patient was not considered in labor.

An attempt was made to give thirty grains of hydrate of chloral, but the dysphagia was so great that only a fractional part of the dose could be given. Seventy grains were then given by the rectum, effectually arresting the convulsions, and producing unconsciousness. The pulse rose to 120 in one hour, when labor pains commenced. A living child was born fourteen hours afterwards. Consciousness did not return until ten hours later, when she for the first time realized that she was a mother.

6. Dr. Saboya states that having found by experience that incisions of the neck of the womb, whether made with the bistoury, the scissors, or the hysterotome, even when followed by the sponge tent, had a tendency to subsequent contraction, he was induced to modify the operation as follows:

A tubular needle armed with a metallic thread, was passed into the cervix uteri one-fifth of an inch from its orifice, and made to emerge at an equal distance on the other side. The wire being then pushed through the needle, its extremity was seized with nippers and held while the needle was withdrawn. A loop was then formed on each extremity of the wire at a distance of about two inches from the cervix. The nippers were then passed into the cervical canal, the middle of the wire seized and drawn down and cut in two. Each of the cut ends of the wire was then passed through the loop of the corresponding side, and slightly twisted. The wires were allowed to remain one month, being twisted a little every eight days. At the end of that time the wires had produced fistulous tracts, the sides of which had no disposition to unite; they were now removed, and the remaining tissues were cut at the same time with a long narrow bistoury guided by a director.

The operation thus described was performed upon two patients, with complete success. A sufficiently patulous os uteri was secured by an almost bloodless operation, and the extremities of the incisions being covered by mucous membrane, had no tendency to become adherent or to contract.

7. Dr. Gallard says, " that while Marion Sims advises washing the hands *before* making an examination, it is thought best in Paris to wash them *afterwards*." Both are right. The hands should be washed both before and after.

